# Features of Idebenone and Related Short-Chain Quinones that Rescue ATP Levels under Conditions of Impaired Mitochondrial Complex I

**DOI:** 10.1371/journal.pone.0036153

**Published:** 2012-04-27

**Authors:** Michael Erb, Barbara Hoffmann-Enger, Holger Deppe, Michael Soeberdt, Roman H. Haefeli, Christian Rummey, Achim Feurer, Nuri Gueven

**Affiliations:** 1 Santhera Pharmaceuticals, Liestal, Switzerland; 2 Biozentrum, University of Basel, Basel, Switzerland; UMASS-Amherst/Tufts University School of Medicine, United States of America

## Abstract

Short-chain quinones have been investigated as therapeutic molecules due to their ability to modulate cellular redox reactions, mitochondrial electron transfer and oxidative stress, which are pathologically altered in many mitochondrial and neuromuscular disorders. Recently, we and others described that certain short-chain quinones are able to bypass a deficiency in complex I by shuttling electrons directly from the cytoplasm to complex III of the mitochondrial respiratory chain to produce ATP. Although this energy rescue activity is highly interesting for the therapy of disorders associated with complex I dysfunction, no structure-activity-relationship has been reported for short-chain quinones so far. Using a panel of 70 quinones, we observed that the capacity for this cellular energy rescue as well as their effect on lipid peroxidation was influenced more by the physicochemical properties (in particular logD) of the whole molecule than the quinone moiety itself. Thus, the observed correlations allow us to explain the differential biological activities and therapeutic potential of short-chain quinones for the therapy of disorders associated with mitochondrial complex I dysfunction and/or oxidative stress.

## Introduction

Quinones, such as coenzyme Q_10_ (CoQ_10_) or vitamin K are a chemical class of compounds containing a quinoid ring system; reviewed by [1], [2] causing them to be involved in a vast range of cellular redox reactions. Importantly, minor variances in their chemical and physicochemical properties can lead to extensive differences in their biological and pharmacological effects but no clear structure-activity relationships (SAR) have been identified so far. Enzymes involved in cellular quinone metabolism catalyze mainly two different redox reactions. NADPH:cytochrome P450 reductases generate semiquinones by incomplete, one-electron reduction [1], [2]. Since semiquinones are rather unstable, there is a high likelihood for this reaction to generate reactive oxygen species (ROS). NAD(P)H:quinone oxidoreductases (NQOs) on the other hand are cytosolic flavoproteins that compete with P450 reductase and catalyze the reduction of quinones and their derivates by complete, two-electron reduction [2]. This process leads to relatively stable hydroquinones, often also referred to as quinols, which does not result in the formation of ROS. Thus, NQOs are considered key detoxifying enzymes, which are induced by stressors such as xenobiotics or oxidants [3]. NQOs have been shown to reduce numerous pharmacologically active compounds such as quinone epoxides, aromatic nitro and nitroso compounds, azo dyes and Cr(VI) compounds [4], with NQO1 showing its highest specificity towards quinones. With respect to benzoquinones, NQOs are able to efficiently reduce CoQ_0_ [5] and CoQ_1_ [6], [7]. These quinones are short-chain analogs of CoQ_10_, which is best known for its pivotal role in mitochondrial oxidative phosphorylation, although the functional significance of NQO-dependent reduction of CoQ_0_ and CoQ_1_ is still unclear.

Idebenone, a benzoquinone carrying exactly the same quinone moiety as CoQ_0_, CoQ_1_ and CoQ_10_, shows multiple activities *in vitro* and *in vivo*. Most prominently associated with idebenone is its potent antioxidant capacity as substantiated by the ability to prevent lipid peroxidation and to protect against ROS-induced damage in multiple systems [8]–[12]. Consistent with this role, idebenone proved cytoprotective after exposure of cultured cells to various toxic insults [8], [10], [11], [13]. Consequently, idebenone is under investigation as a possible treatment for disorders characterized by excessive oxidative damage due to mitochondrial defects. Idebenone is quickly absorbed and is well tolerated and safe [14]. Successful treatment of a patient with Leigh syndrome using idebenone, where high-dose CoQ_10_ had no effect on respiratory function, is indicative of therapeutic levels of idebenone in the brain [15]. Thus, idebenone has been suggested for treating patients with mitochondrial diseases, such as *mitochondrial encephalopathy, lactic acidosis and stroke-like episodes* (MELAS) [16], [17] and *Leber's hereditary optic neuropathy* (LHON) [18], [19].

When Tsuruo *et al* [20] reported that in brain tissue of idebenone-treated mice NQO1 activity was increased in a dose-dependent manner, we studied this interaction in more detail [21]. We observed that idebenone and other short-chain quinones, but not the structurally related long-chain quinone CoQ_10_, are excellent substrates for reduction by NQO1 [21]. Importantly, some of these NQO1-reduced quinones are able to donate electrons into the mitochondrial respiratory chain to rescue cellular adenosine triphosphate (ATP) levels under conditions of impaired complex I function [6], [21], [22]. Consistent with this cytoplasmic-mitochondrial redox cycling hypothesis, these quinones also reduced extracellular lactate levels in a cell culture model of MELAS.

Here, we compared a set of 70 short-chain quinone derivatives with structurally modified quinone units or chain moieties. Using this number of compounds, clear correlations of their logD values with their ability to rescue ATP levels and activity to suppress lipid peroxidation emerge, that connect physicochemical parameters to their respective biological activities.

## Results

Under conditions of dysfunctional complex I, it is well described that certain short-chain quinones are capable of generating ATP by a cytoplasmic-mitochondrial electron transfer [6], [21], [22]. This electron transfer involves the reduction of quinones by cytoplasmic NQO1 or related reductases, which is associated with a reduction of cellular NADH levels [21]. Upon translocation to the mitochondria these quinones are able to donate their electrons to complex III in a antimycin-sensitive manner to increase mitochondrial membrane potential, sustain mitochondrial respiration and ATP synthesis in the presence of the complex I inhibitor rotenone [6], [21], [22]. Not surprising, the effect of cellular rotenone exposure on ATP levels is highly dependent on cell culture medium glucose levels (Figure S1A) since under conditions of high glucose cellular energy is predominantly generated by glycolysis. We observed that the reduction of cellular ATP levels by rotenone occurs within minutes under low glucose conditions (Figure S1B). However, since only 5 minutes of incubation with quinones is sufficient to significantly restore ATP levels after conditions of prolonged (55 minutes) rotenone-induced ATP deficiency [21], it can be assumed that during the time interval before quinones are added no major cell death is induced by the rotenone treatment itself.

**Figure 1:**
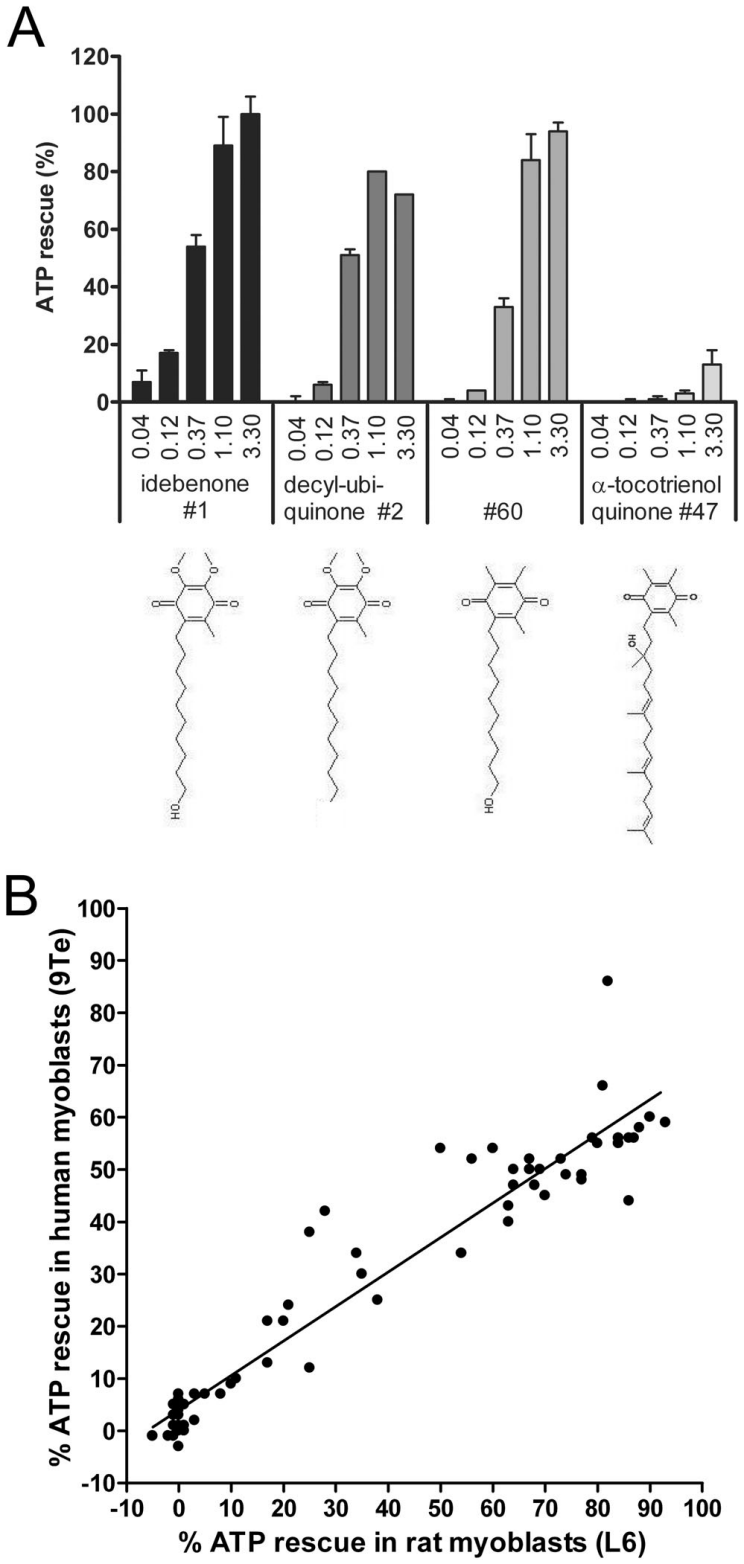
Efficacy of ATP rescue in the presence of rotenone by different quinones. (A) ATP rescue by 4 different quinones was determined in rotenone-treated rat myoblasts (L6). ATP rescue is defined as the percentage of quinone-induced increase in ATP levels in the presence of rotenone, relative to the ATP reduction by rotenone alone. Bars represent the mean of 3 wells, error bars represent standard deviation. (B) ATP rescue by different quinones is not cell type- or species-dependent. ATP rescue by 70 quinones was determined in primary human myoblasts (9Te), rat myoblasts (L6) and human immortalized hepatic cells (HepG2). Correlation of values derived from rat myoblasts (L6) and human myoblasts (9Te), are shown (R^2^ = 0.8434). Similar results were obtained for rat myoblasts (L6) vs. human immortalized hepatic cells (HepG2) as well as human myoblasts (9Te) vs. human immortalized hepatic cells (HepG2) (Figure S1). For reasons of clarity, error bars were omitted from (B) but standard deviation (SD) values can be found in Supplementary Table S2 which lists all results.

Since so far, no systematic correlation of the chemical nature of quinones to their activity profile was identified, it was of interest to elucidate possible structure-activity relationships (SAR) using a diverse set of compounds. Before testing multiple quinones for this activity however, it was important to determine if the concentration range for the described ATP-rescue effect would differ significantly from compound to compound. For this purpose, four quinones were selected that differed either in their quinone moiety or the structure of the tail. When these quinones were tested at concentrations from 0.04 to 3.3 µM it became evident that most of them dose-dependently rescued ATP levels *in vitro*, with a maximum effect at concentrations from 1 to 3.3 µM (Figure 1). While idebenone (#1), decyl-ubiquinone (#2) and compound #60 showed largely comparable dose-effect relationships, α-tocotrienol quinone (#47) only showed a minor effect (10% rescue) at the highest concentration (Figure 1). Strikingly, when comparing the structures of the compounds it appeared that the quinone moiety itself appeared to have less of an effect on the capacity to rescue ATP levels compared to the effect of the lipophilic tail of the compound. Decyl-ubiquinone (#2) for example, which differs from idebenone (#1) only by the absence of the terminal hydroxyl group of the tail, showed 30% reduced activity at 3.3 µM compared to idebenone while at other concentrations no differences could be detected. Even more striking, when comparing compound #60 with α-tocotrienol quinone (#47), which harbor the identical quinone moiety, a significant difference in activity was evident at all concentrations tested (Figure 1).

We then synthesized or acquired 70 related quinone compounds, which largely clustered into nine structural groups that for example contained structural motifs of idebenone, CoQ_10_, vitamin E or vitamin K (Table S1). These compounds were tested for their ability to rescue ATP levels in three different cell lines/strains to address the reliability of the assay with regards to the species and tissue from which the cells were derived. Comparison of results derived from primary human myoblasts (9Te) against the activity found in a rat myoblast cell line (L6) showed a very good correlation (Figure 1B, R ^2^ = 0.8434). Similarly, when comparing myoblast cells (9Te, L6) with the human immortalized hepatic cell line HepG2, equally good correlations were obtained (Figure S1C and D), which suggested that the capacity of quinones to rescue ATP levels under conditions of impaired complex I were not dependent on particular species or tissues. Thus, for all subsequent results only data from L6 cells are shown, while the data for the other two cell lines (9Te, HepG2), which were generally very similar, can be found in the supplementary figures (Figures S1, S2, S3).

**Figure 2:**
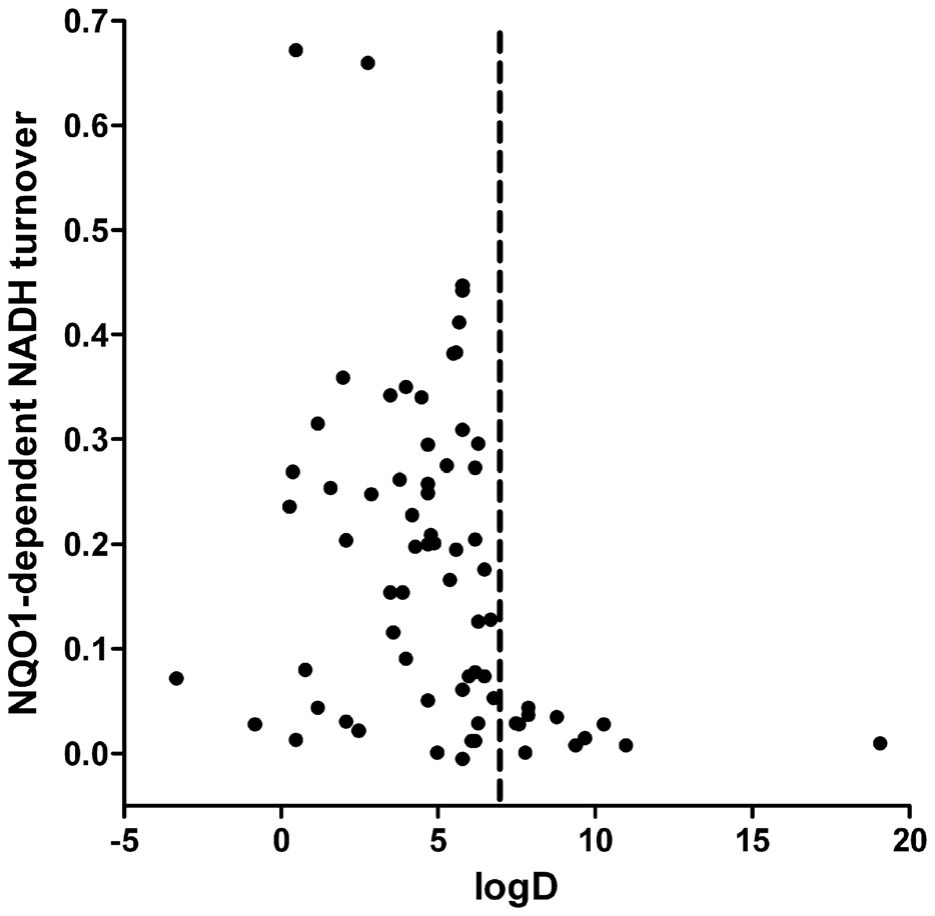
Reduction of quinones by NQO1 in a cell free assay system is dependent on their logD values. *In vitro* reduction of 70 quinone derivatives by recombinant NQO1 was correlated to their calculated logD values. Each data point represents the mean from 2 independent experiments (see Table S1 for details about the quinones used). For reasons of clarity, error bars were omitted from graphs but standard deviation values can be found in Supplementary Table S2 which lists all results.

The results in Figure 1A suggested that structural modifications to the chain, which determined the physicochemical properties to a major extent, were influencing the ATP rescue activity, rather than modifications in the quinone moiety itself. In addition, previous work implied that this ATP rescue activity depends on the prior reduction of quinones, for example by NQO1 [6], [21]. Therefore, we compared the reduction of this set of quinones by recombinant NQO1 enzyme *in vitro* against their logD value, which is an indicator of their hydrophilicity/lipophilicity (Figure 2). The results demonstrate that not all quinones were equally reduced by recombinant NQO1 in a cell-free environment. More importantly though, a strict cut-off was evident (dashed line) at around a logD value of 7, above which the reduction of quinones by NQO1 was negligible, while below this threshold, little dependence between logD and reduction by NOQ1 could be detected.

**Figure 3:**
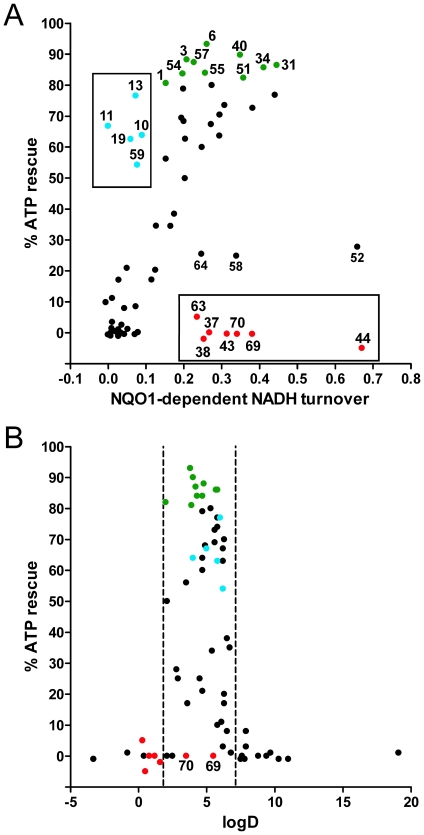
Cellular ATP rescue by different quinones in the presence of rotenone is dependent on their logD value. (A) Cellular ATP rescue capacity was correlated to the rate of reduction by recombinant NQO1 enzyme in a cell-free assay. Clear outliers (boxed compounds, red circles) are either mitochondrially targeted (#69, 70) or have logD values <2. (Compare to Figure 3B). Similarly, some compounds demonstrated good ATP rescue activity while being poorly reduced by NQO1 in vitro (boxed compounds, blue circles). Of all compounds tested, about 14% demonstrated very good (>80%) ATP rescue activity (green circles). (B) ATP rescue ability was correlated to the logD value of each compound. Data represent values for rat myoblasts (L6). Similar results were obtained for human immortalized hepatic cells (HepG2) and human myoblasts (9Te) (Figure S2). The data represent one typical experiment out of three experiments, which yielded similar results. The values are means ± SD, n = 3 replicate wells. Color coding of results is analogous to (A). For reasons of clarity, error bars were omitted from graphs but standard deviation values can be found in Table S2, which lists all results.

Although previous reports indicated, using a limited number of quinones, that the ATP rescue activity was strictly dependent on reduction by NQO1 in cells [6], [21], no clear correlation between the reduction by NQO1 in a cell-free assay system and cellular ATP rescue activity was found (Figure 3A). In fact, some compounds (#37, 38, 43, 44, 63, 69, 70), despite a significant turnover by NQO1, completely failed to protect against rotenone-induced loss of ATP (Figure 3A, boxed red circles). At the same time some compounds (# 10, 11, 13, 19, 59) showed significant ATP-rescue activity in cells but hardly any reduction by NQO1 in cell free assays (Fig. 3A, boxed blue circles). This discrepancy suggested that other parameters in addition to the reduction by NQO1 also influence the ability to rescue ATP levels under conditions of impaired complex I. Surprisingly, when ATP rescue activities were correlated to the logD values of the quinones (Figure 3B), it emerged that in addition to the cut-off observed for the cell-free assay at a logD value of around seven (Figure 2), a second cut-off at a logD value of around two was detected (dashed lines). All quinones with a logD of less than 1.9 and the mitochondria-targeted compounds (#69, 70) were unable to rescue ATP levels in rat myoblasts (L6) cells, despite being strongly reduced by NQO1 in a cell-free system (Figure 3A boxed compounds; Figure 3B red circles). Very similar results with regards to logD-cut off values were also observed in human hepatic cells (HepG2) and human myoblasts (9Te) (Figure S2).

**Figure 4:**
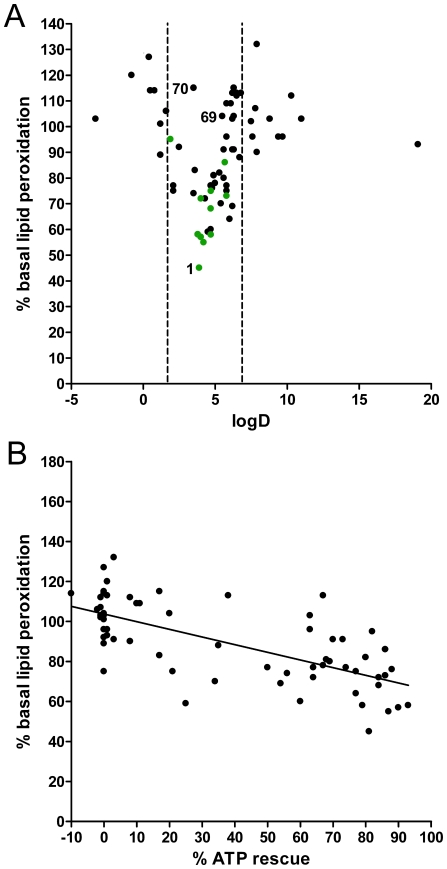
Effect of quinones on basal lipid peroxidation is dependent on their logD value. (A) Cellular levels of basal lipid peroxidation were measured using BODIPY-C_11_ dye and correlated with the respective logD value. Response to DMSO was set to 100%. Compounds #69 and 70 represent two mitochondrially targeted idebenone molecules. The values are means ± SD, n = 3 replicate wells. (B) Lipid peroxidation values were correlated to ATP rescue efficiency of each quinone. Data represent values for L6 cells. Similar results were obtained for human immortalized hepatic cells (HepG2) and human myoblasts (9Te) (Figure S3). For reasons of clarity, the highly prooxidative (>200%) compounds (# 36,44,52,63 64) and error bars were omitted from graphs but standard deviation values can be found in Supplementary Table S2, which lists all results.

Some short chain quinones such as idebenone are described to be potent antioxidants that prevent lipid peroxidation in multiple systems [8]–[12], although at the same time some reports claim that they can also act as pro-oxidants [23], [24]. We therefore investigated if the quinones described here also affected basal levels of lipid peroxidation using the ratiometric fluorescent dye BODIPY-C_11_ and compared it against their logD properties (Figure 4A). Strikingly, when correlating basal levels of lipid peroxidation against the logD values, we observed a pattern that inversely mirrored the results of the ATP rescue/logD correlation (Figure 3B). In the same logD window (2<logD>7), basal levels of lipid peroxidation were significantly reduced, with idebenone (#1) as the most potent of the 70 quinones reducing levels by more than 50%. Analogous to the results for ATP rescue (Figure 3B) the mitochondria-targeted quinones (#69, 70) were also unable to reduce basal levels of lipid peroxidation (Figure 4A). Despite an antioxidant effect by the majority of quinones, clearly some compounds increased levels of lipid peroxides, with the highest levels observed from compounds outside the permissive logD range (e.g. #17, 37, 62). However, there was no clear indication that the pro-oxidative profile correlated with their logD values. Similar results were also observed in human hepatic cells (HepG2) and human primary myoblasts (9Te) (Figure S3). As suggested by the similarities of the observed logD ranges in Figures 3B and 4A, an inverse correlation (R^2^ = 0.4709) between the ATP rescue activity and basal levels of lipid peroxidation could be demonstrated in rat myoblasts (L6) (Figure 4B) as well as in human myoblasts (9Te) and human hepatic cells (HepG2) (data not shown).

## Discussion

Bioactivation of pharmacophores by NQO1 has been described by multiple studies; reviewed by [25]. Most prominently, β-lapachone and mitomycin C exhibit their biological activity only upon their NQO1-dependent reduction [26], [27]. For both of these compounds however, bioactivation by NQO1 is associated with a toxic gain of function. In contrast, for some-short chain quinones described here, bioactivation is associated with a protection of cellular ATP levels under conditions of impaired complex I function [6], [21], [22] and reduced levels of lipid peroxidation. To demonstrate this effect, previous studies used quinone concentrations, which were much higher than concentrations that can safely be achieved *in vivo* (high µM range) [8], [22]. Here, we describe for the first time that this mechanism can be observed *in vitro* at physiological concentrations, even in the low nanomolar range, which makes short-chain quinones an attractive option for the treatment of disorders associated with impaired mitochondrial complex I such as *mitochondrial encephalopathy, lactic acidosis and stroke-like episodes* (MELAS) [16], [17] and *Leber's hereditary optic neuropathy* (LHON) [18], [19]. Although NQO1-dependent electron shuttling from the cytoplasm to the mitochondria by quinones has been described previously [6], [21], [22], it was unclear whether these reports could be seen as individual observations that are exclusively linked to certain cellular model systems. Furthermore, no information about a possible structure-activity relationship of these quinones was available. The results described here indicate, that this particular electron shuttling process and the resulting rescue of ATP levels are not species or tissue specific responses but instead appear to represent a mechanism found in multiple organisms, cells and tissues. Furthermore, we described for the first time that this effect of short-chain quinones seems to be governed more by intrinsic physicochemical properties of the whole molecule, such as the lipophilicity, rather than by the specific structural features or the redox potential of the quinone moiety alone.

Not unexpectedly, we observed an inverse correlation between the ATP rescue activity by different quinones and their effect on basal levels of cellular lipid peroxidation with almost identical windows of permissive logD values. This observation could indicate that the described activity of idebenone against lipid peroxidation [8]–[12] is dependent on its physicochemical characteristics and could therefore also apply to other quinones. Consistent with our observations, Chan *et al.* [6] also described that the ability of CoQ_1_ (#39) to rescue ATP levels is associated with a protection of cellular glutathione (GSH) levels, thus the capacity to rescue ATP levels appears to be tightly linked to the cellular antioxidant capacity. Evidence that short-chain quinones can only act as antioxidants in the reduced state [28] implies that for dietary short-chain quinones to act as antioxidants, an efficient reduction by cytoplasmic NQO1 or by other reductases has to be a prerequisite. At the same time, these quinones also have to be sufficiently lipophilic to enter cellular membranes and thus prevent lipid peroxidation. And finally, quinones that continuously shuttle between cytoplasmic reduction and oxidation within membranes could act catalytically. Such a mechanism would make them highly superior to radical scavengers that are mechanistically only able to detoxify radicals in an equimolar ratio. In support of this, it has to be noted that two of the quinones tested, were localized to the mitochondria by attaching a specific targeting peptide (#69, #70). These quinones, unable to shuttle between cytoplasm and mitochondria, did not rescue ATP levels and also did not lower basal levels of lipid peroxidation, despite having a permissive logD value and consequently being efficiently reduced by NQO1. The limited solubility in aqueous buffer of the more hydrophobic quinones could account for the lower rates of reduction by NQO1. For this purpose we have previously formulated the most hydrophobic quinone of the quinones tested here, CoQ10 (#42) by two methods commonly used (complexed with BSA and formulated into liposomes) [21]. Under both conditions CoQ10 was not reduced by recombinant NQO1 in a cell free assay, while at the same time idebenone was efficiently reduced. Thus, the balance between hydrophilicity, which controls the efficient cytoplasmic reduction by NQO1, and lipophilicity, which determines the access to membranes is likely to be essential for the observed rescue of ATP levels and the reduction of lipid peroxidation. Interestingly, some of the most potent compounds for reducing lipid peroxidation (#1, 39) are also reported to display complex I inhibitory activity [29]–[31]. In particular it has been proposed that idebenone (#1) in the reduced state has a very slow “off” rate from complex I and thus blocks the CoQ_10_ binding site [32]. The authors also suggested that idebenone can additionally interact with the NADH binding site of complex I exposed to the mitochondrial matrix. Whether these activities on complex I contribute to the antioxidant function of short chain quinones and how the two protective effects of short chain quinones, ATP rescue and antioxidant function are connected in detail will have to be elucidated in future experiments.

For our SAR investigations regarding the physicochemical properties of short-chain quinones, we also looked at a potential relationship between their biological effects (reduction by NQO1, ATP rescue and protection against lipid peroxidation) and the pKa values in addition to their logD values. However, based on current results from the limited number of quinones with a basic center, no clear correlation could be demonstrated, which warrants further studies using additional quinone derivatives specifically designed to compare compounds with similar logD values but different pKa values.

**Figure 5:**
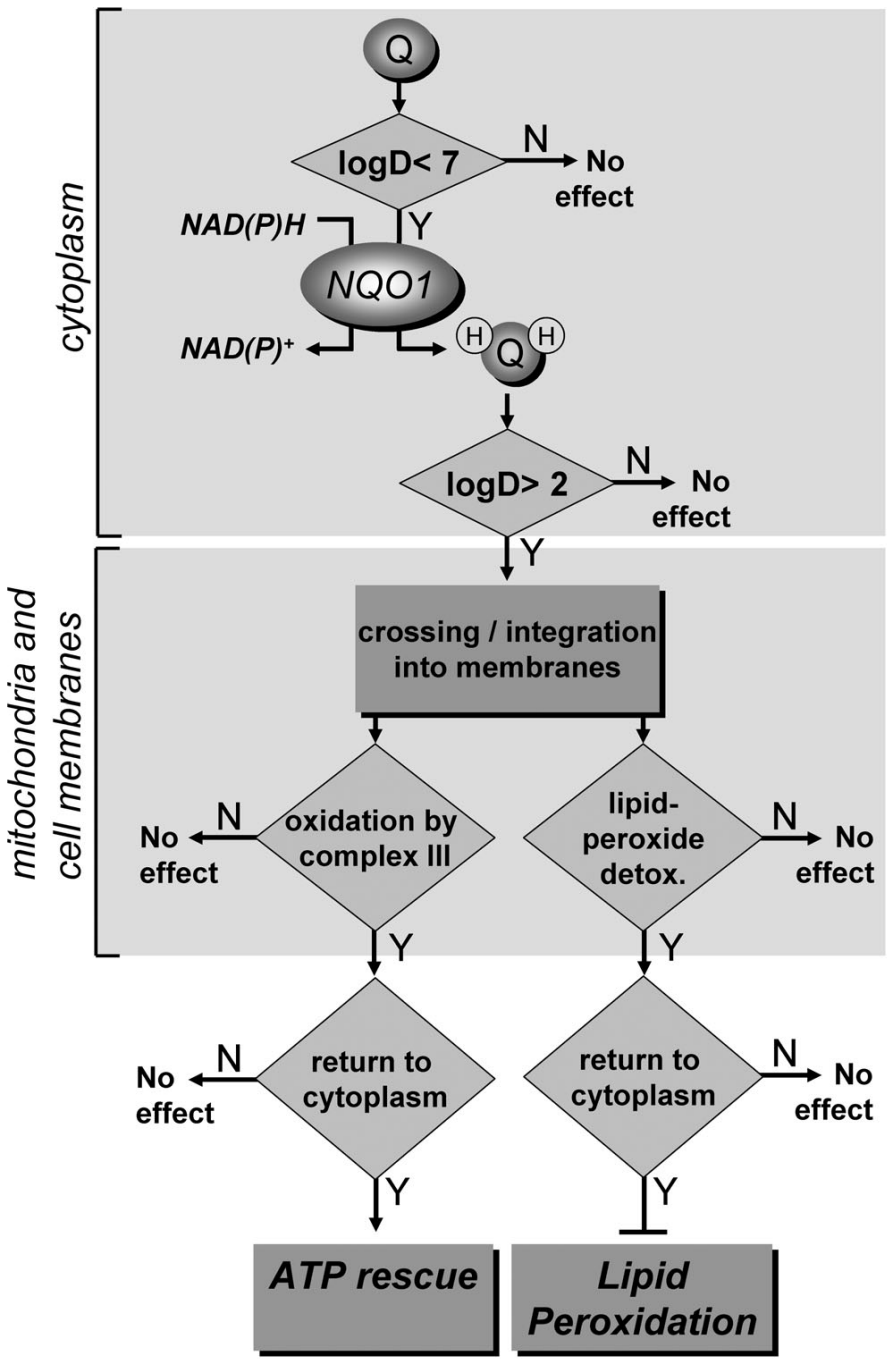
Schematic representation of the requirements for cytoplasmic-mitochondrial electron transfer and ATP rescue. Only quinones with a logD<7 are reduced by cytoplasmic NQO1 or other reductases. Once reduced, and depending on permissive lipophilicity characteristics (2<logD>7), they can shuttle electrons into the mitochondrial respiratory chain by reducing complex III or integrate into lipid membranes to prevent lipid peroxidation. However, these effects manifest only, if the compounds upon oxidation can return to the cytoplasm to be reduced again, which allows them to act in a catalytical manner. (N: no, Y: yes).

In summary, the results described here imply that the ATP rescue ability of short chain quinones, under conditions of defective mitochondrial complex I, is strongly determined by the hydrophilicity/lipophilicity (logD) of the entire molecule rather than particular structural features. This hydrophilicity/lipophilicity profile in turn determines the reduction by NQO1, influences levels of lipid peroxides by their antioxidant function and finally governs their interaction with the electron transport chain of the mitochondria. The model described here, rationalizes for the first time the connection of physicochemical characteristics of short-chain quinones with their biological activities in cells and tissues (Figure 5). Importantly, it also explains why only a very limited number of short-chain quinones, such as idebenone, that combine a specific set of favorable characteristics can be considered beneficial for the treatment of mitochondrial disorders that are associated with increased levels of ROS and reduced energy supply.

## Materials and Methods

### Reagents and Chemicals

All chemical reagents, if not otherwise stated, were purchased from Sigma (Sigma-Aldrich, Buchs, Switzerland). All cell culture media and solutions, if not otherwise stated, were purchased from PAA (Pasching, Austria). Most quinones were synthesized in-house with a purity of ≥95% as determined by NMR and LCMS. The synthetic pathways as well as the synthetic preparation procedures for the test compounds listed in Table S1 of this paper were described previously [33], [34]. For all assays described, compounds were dissolved at 10 mM (stock solution) in 100% DMSO (Acros Organics, Belgium). Predicted LogD values (at pH 7.4) were calculated using ACD/Labs software, (Frankfurt, Germany; Version 12; ACD/Labs 1994–2009). The model implemented in the software is derived from a large amount of experimental data and allows sound extrapolations towards unknown compounds. Details on the database can be found on the ACD/Labs homepage.

### Cell culture

Rat myoblast cells (L6, CRL-1458, ATCC, Molsheim, France) and human hepatic cells (HepG2, 330198, CLS, Eppelheim, Germany) were cultured under standard conditions (37°C, 5% CO_2_, 90% humidity; DMEM, 1 g/l Glucose, 10% fetal bovine serum (FBS), Penicillin-Streptomycin-Glutamine). Primary human myoblasts (9Te) [35] were cultured in MEM EBS supplemented with 25% M-199 EBS, 10% Hyclone FCS, 10 µg/ml insulin, 100 ng/ml EGF, 100 ng/ml FGF and Penicillin-Streptomycin-Glutamine as described previously [35] and under conditions described above. Significant NQO1 expression, necessary for NQO1-dependent reduction of quinones, has been described previously for myoblasts and HepG2 cells [21].

### NQO1 dependent reduction of quinones

Reduction of quinones by recombinant NQO1 (Sigma, Buchs, Switzerland) was measured essentially according to a modified protocol by Ernster [36]. Reactions were performed in 1-ml disposable cuvettes at room temperature in reaction buffer (25 mM Tris-HCl pH 7.4, 0.7 mg/ml bovine serum albumin (BSA), 1 µg/ml enzyme, 10 µM flavin adenine dinucleotide (FAD), 50 µM quinone). Before the reaction was started by addition of 200 µM NADH, the basal absorbance of the quinone containing reaction solution was measured in a spectrophotometer (Ultrospec® 3000, Amersham Pharmaceutical Biotech, Little Chalfont, UK). Upon the addition of NADH, the NADH absorbance (340 nM) was measured during 60 seconds. The decrease in NADH was estimated using the following calculation: A_basal_+A_200 µM NADH_−A_60 sec_. The assay was performed in duplicates and the mean was used for correlations.

### Quinone-dependent rescue of ATP levels

Cells were seeded at a density of 10^4^ cells per well in a 96-well plate in the corresponding culture medium (containing 1 g/l Glucose) one day before the experiment. Cells were treated with quinones in presence of rotenone (1 µM) for 60 minutes in DMEM without glucose. Subsequently, cells were lysed and ATP levels were determined as described previously [23]. Cellular ATP levels were quantified using luminescence from the ATP-dependent enzymatic oxidation of luciferin by luciferase. Briefly, cells were lysed in a volume of 100 µl (4 mM EDTA, 0.2% Triton X-100) for five minutes. In 96-well plates, 100 µl of ATP measurement buffer (25 mM HEPES pH 7.25, 300 µM D-luciferin, 5 µg/ml firefly luciferase, 75 µM DTT, 6.25 mM MgCl_2_, 625 µM EDTA and 1 mg/ml BSA) was combined with 10 µl lysate to start the reaction. Luminescence was quantified immediately using a multimode plate reader (Tecan M1000, Tecan iControl 1.6 software; Tecan Austria GmbH, Grödig, Austria). ATP rescue is defined as the percentage of quinone-induced increase in ATP levels in presence of rotenone relative to the ATP reduction by rotenone alone. The data represent one typical experiment out of three experiments, which yielded similar results. The values are means ± SD, n = 3 replicate wells.

### Quantification of basal levels of lipid peroxidation

To assess basal levels of lipid peroxidation, cells were seeded at a density of 2×10^4^ cells per well in black 96-well plates (Greiner, Frickenhausen, Germany) in the corresponding culture medium one day before the experiment. Cells were preincubated with BODIPY 581/591 C_11_ (D3861 Invitrogen, Karlsbad, USA) by replacing the medium with 50 µl HBSS containing 1‰ BODIPY 581/591 C_11_ for 30 minutes. The preincubation solution was replaced with 100 µl HBSS followed by the addition of the quinones. After one hour of quinone treatment, the cells were washed three times with PBS and BODIPY fluorescence was acquired immediately (Ex/Em 490/520 nM and 490/600 nM), using a multimode plate reader. From the measured intensities, the background intensity from control wells without cells was subtracted before the ratio of green to red fluorescence was calculated. The ratio obtained from DMSO treated cells was set to 100% and represents the level of basal lipid peroxidation. Due to the known absorption characteristics for some short chain quinones at 279 nm it is very unlikely that quinone-dependent quenching can take place in this assay. Nevertheless, especially for novel uncharacterized compounds, this possibility can not be absolutely excluded.

## Supporting Information

Figure S1 **Figure S1A: HepG2 cells were incubated for 1 hour with various concentrations of glucose (0.0 to 3.2 g/l glucose).** (A) The effect of rotenone treatment (6 µM) on cellular ATP levels was evaluated for each glucose concentration. ATP levels are expressed as percentage of DMSO-treated cells under conditions of 3.2 g/l glucose. Cell culture medium glucose levels did only affect the ATP levels in presence of rotenone. Bars represent mean+SD of triplicates of one typical out of two independent experiments. (B) Figure S1B: HepG2 cells were incubated for 1 hour in glucose-free DMEM. Rotenone was added at various time points to a final concentration of 6 µM. ATP levels are expressed as percentage of the ‘0 minute rotenone’ treated control cells (white bar). Rotenone exposure reduced ATP levels in a time dependant manner. Bars represent mean+SD of triplicates of one typical out of two independent experiments. (C) Comparison of ATP rescue capacity of 70 quinone derivatives comparing the effects in human immortalized hepatic cells (HepG2) versus human myoblasts (9Te) (D) Comparison of ATP rescue capacity of 70 quinone derivatives comparing the effects in human immortalized hepatic cells (HepG2) versus rat myoblasts (L6) (lower panel). Data is expressed as percent ATP rescue (DMSO = 0%). For reasons of clarity, error bars were omitted but standard deviation values can be found in Supplementary Table S2, which lists all results.
(TIF)

Figure S2 **Correlation of ATP rescue capacity against logD values of 70 quinone derivatives comparing the effects in human immortalized hepatic cells (HepG2) (upper panel) and human myoblasts (9Te) (lower panel).** The data represent one typical experiment out of three experiments, which yielded similar results. The values are means ± SD, n = 3 replicate wells. For reasons of clarity, error bars were omitted but standard deviation values can be found in Supplementary Table S2, which lists all results.
(TIF)

Figure S3 Correlation of the effect on basal levels of lipid peroxidation against logD values of 70 quinone derivatives in human immortalized hepatic cells (HepG2) (upper panel) and human myoblasts (9Te) (lower panel). Response to DMSO was set to 100%. The values are means ± SD, n = 3 replicate wells. For reasons of clarity, error bars were omitted but standard deviation values can be found in Supplementary Table S2, which lists all results.
(TIF)

Table S1 **List of quinones tested.**
(DOC)

Table S2 **List of all data generated for 3 cell lines and all assays.** The following cell lines/strains were used: human immortalized hepatic cells (HepG2), human myoblasts (9Te) and rat myoblasts (L6).
(XLS)

## References

[1] O'Brien PJ (1991). Molecular mechanisms of quinone cytotoxicity.. Chem Biol Interactions.

[2] Monks TJ, Hanzlik RP, Cohen GM, Ross D, Graham DG (1992). Contemporary issues in toxicology.. Toxicol Appl Pharmacol.

[3] Long DJ, Jaiswal AK (2000). NRH:quinone oxidoreductase2 (NQO2).. Chem Biol Interact.

[4] Colucci MA, Moody CJ, Gouch GD (2008). Natural and synthetic quinones and their reduction by the quinone reductase enzyme NQO1: from synthetic organic chemistry to compounds with anticancer potential.. Org Biomol Chem.

[5] Boutin, JA Chatelain-Egger F, Vella F, Delagrangea P, Ferry G (2005). Quinone reductase 2 substrate specificity and inhibition pharmacology.. Chem Biol Interact.

[6] Chan TS, Teng S, Wilson JX, Galati G, Khan S (2002). Coenzyme Q cytoprotective mechanisms for mitochondrial complex I cytopathies involves NAD(P)H: quinone oxidoreductase 1(NQO1).. Free Radic Res.

[7] Dragan M, Dixon SJ, Jaworski E, Chan TS, O'Brien PJ (2006). Coenzyme Q(1) depletes NAD(P)H and impairs recycling of ascorbate in astrocytes.. Brain Res.

[8] Suno M, Akinobu N (1984). Inhibition of lipid peroxidation by a novel compound (CV-2619) in brain mitochondria and mode of action of the inhibition.. Biochem Biophys Res Comm.

[9] Sugiyama Y, Fujita T, Matsumoto M, Okamoto K, Imada I (1985). Effects of idebenone (CV-2619) and its metabolites on respiratory activity and lipid peroxidation in brain mitochondria from rats and dogs.. J Pharmacobio-Dyn.

[10] Suno M, Akinobu N (1989). Inhibition of lipid peroxidation by idebenone in brain mitochondria in the presence of succinate.. Arch Gerontol Geriatr.

[11] Rauchovà H, Vrbacky M, Bergamini C, Fato R, Lenaz G (2006). Inhibition of glycerophosphate-dependent H_2_O_2_ generation in brown fat mitochondria by idebenone.. Biochem Biophys Res Comm.

[12] Ranganathan S, Harmison GG, Meyertholen K, Pennuto M, Burnett BG (2009). Mitochondrial abnormalities in spinal and bulbar muscular atrophy.. Hum Mol Genet.

[13] Jauslin ML, Wirth T, Meier T, Schoumacher F (2002). A cellular model for Friedreich Ataxia reveals small-molecule glutathione peroxidase mimetics as novel treatment strategy. Human Mol.. Genet.

[14] Becker C, Bray-French K, Drewe J (2010). Pharmacokinetic evaluation of idebenone. Expert Opin Drug Metab Toxicol..

[15] Haginoya K, Miyabayashi S, Kikuchi M, Kojima A, Yamamoto K (2009). Efficacy of idebenone for respiratory failure in a patient with Leigh syndrome: a long-term follow-up study.. J Neurol Sci.

[16] Ikejiri Y, Mori E, Ishii K, Nishimoto K, Yasuda M (1996). Idebenone improves cerebral mitochondrial oxidative metabolism in a patient with MELAS.. Neurology.

[17] Napolitano A, Salvetti S, Vista M (2000). Long-term treatment with idebenone and riboflavin in a patient with MELAS.. Neurol Sci.

[18] Klopstock T, Yu-Wai-Man P, Dimitriadis K, Rouleau J, Heck S (2011). A randomized placebo-controlled trial of idebenone in Leber's hereditary optic neuropathy.. Brain 134(Pt.

[19] Carelli V, La Morgia C, Valentino ML, Rizzo G, Carbonelli M (2011). Idebenone treatment in Leber's hereditary optic neuropathy.. Brain 134(Pt.

[20] Tsuruo Y, Ishimura K, Tamura M, Kagawa S, Morita K (1994). Biochemical and histochemical studies of the effects of cerebral metabolism-improving drugs on NADPH diaphorase activity in mouse brain. Jpn J Pharmacol..

[21] Haefeli RH, Erb M, Gemperli AC, Robay D, Courdier Fruh I (2011). NQO1-dependent redox cycling of idebenone: effects on cellular redox potential and energy levels. PLoS One.. 2011 Mar 31;.

[22] Giorgio V, Petronilli V, Ghelli A, Carelli V, Rugolo M (2011). The effects of idebenone on mitochondrial bioenergetics. Biochim Biophys Acta..

[23] King MS, Sharpley MS, Hirst J (2009). Reduction of hydrophilic ubiquinones by the flavin in mitochondrial NADH:ubiquinone oxidoreductase (Complex I) and production of reactive oxygen species. Biochemistry..

[24] Genova ML, Pich MM, Biondi A, Bernacchia A, Falasca A (2003). Mitochondrial production of oxygen radical species and the role of Coenzyme Q as an antioxidant. Exp Biol Med (Maywood)..

[25] Ross D, Kepa JK, Winski SL, Beall HD, Anwar A (2000). NAD(P)H:quinone oxidoreductase 1 (NQO1): chemoprotection, bioactivation, gene regulation and genetic polymorphisms. Chem Biol Interact..

[26] Pink JJ, Planchon SM, Tagliarino C, Varnes ME, Siegel D (2000). NAD(P)H:Quinone oxidoreductase activity is the principal determinant of β-lapachone cytotoxicity.. J Biol Chem.

[27] Adikesavan AK, Barrios R, Jaiswal AK (2007). In vivo role of NAD(P)H:Quinone oxidoreductase 1 in metabolic activation of mitomycin C and bone marrow cytotoxicity.. Cancer Res.

[28] Mordente A, Martorana GE, Minotti G, Giardina B (1998). Antioxidant properties of 2,3-dimethoxy-5-methyl-6-(10-hydroxydecyl)-1,4-benzoquinone (idebenone). Chem Res Toxicol..

[29] Degli Esposti M, Ngo A, Ghelli A, Benelli B, Carelli B (1996). The interaction of Q analogs, particularly hydroxydecyl-benzoquinone (idebenone), with the respiratory complexes of heart mitochondria.. Arch Biochem Biophys.

[30] Fato R, Bergamini C, Bortolus M, Maniero AL, Leoni S (2008). Differential effects of mitochondrial Complex I inhibitors on production of reactive oxygen species.. Biochim Biophys Acta.

[31] Rauchová H, Drahota Z, Bergamini Z, Fato R, Lenaz G (2008). Modification of respiratory-chain enzyme activities in brown adipose tissue mitochondria by idebenone (hydroxydecyl-ubiquinone).. J Bioenerg Biomembr.

[32] King MS, Sharpley MS, Hirst J (2009). Reduction of hydrophilic ubiquinones by the flavin in mitochondrial NADH:ubiquinone oxidoreductase (Complex I) and production of reactive oxygen species. Biochemistry..

[33] Feurer A, Gueven N, Hoffmann-Enger B, Erb M, Deppe H (2012). Benzoquinone Derivatives as Modulators of Mitochondrial Function.. WO/2012/.

[34] Feurer A, Hoffmann-Enger B, Deppe H, Soeberdt M, Gueven N (2012). Novel Benzoquinone Derivatives and Use thereof as Modulators of Mitochondrial Function WO/2012/.

[35] Courdier-Fruh I, Briguet A (2006). Utrophin is a calpain substrate in muscle cells.. Muscle Nerve;.

[36] Ernster L (1967). DT-diaphorase.. Methods Enzymol.

