# Supplementary material for: Features of Idebenone and Related Short-Chain Quinones that Rescue ATP Levels under Conditions of Impaired Mitochondrial Complex I
Source: PLoS One. 2012 Apr 27;7(4):e36153. doi: 10.1371/journal.pone.0036153 (PMC3338594; doi:10.1371/journal.pone.0036153)
Supplement: Table S1 — List of quinones tested. (DOC) [file pone.0036153.s004.doc]

**Group 1:** Idebenone-type quinones [33, 34]

**1 – 7 8 – 36**

| **Cpd** | **Compound name / type** | **X** | **Y** | **R1** |
| --- | --- | --- | --- | --- |
| **1** | Idebenone | (CH_2_)_10_ | - | OH |
| **2** | Decyl-Q | (CH_2_)_10_ | - | H |
| **3** |  | (CH_2_)_9_ | - | 3-Me-1,2,4-oxadiazol-5-yl |
| **4** |  | (CH_2_)_10_ | - | pyrrolidin-1-yl |
| **5** |  | (CH_2_)_10_ | - | 1-(4-N-methyl)-piperazinyl |
| **6** |  | (CH_2_)_10_ | - | 1-morpholinyl |
| **7** |  | (CH_2_)_10_ | - | 1-(4-F)-piperidinyl |
| **8** |  | (CH_2_)_9_ | CO | OH |
| **9** |  | (CH_2_)_9_ | CO | NEt_2_ |
| **10** |  | (CH_2_)_9_ | CO | 1-pyrrolidinyl |
| **11** |  | (CH_2_)_10_ | NH | CH_2_CF_3_ |
| **12** |  | (CH_2_)_10_ | NH | phenyl |
| **13** |  | (CH_2_)_10_ | NH | 2-pyridyl |
| **14** |  | (CH_2_)_10_ | NH | 3-pyridyl |
| **15** |  | (CH_2_)_10_ | O | phenyl |
| **16** |  | (CH_2_)_10_ | O | 4-Cl-phenyl |
| **17** |  | (CH_2_)_10_ | O | 3-Cl-4-F-phenyl |
| **18** |  | (CH_2_)_10_ | O | 3-(trifluoromethyl)-phenyl |
| **19** |  | (CH_2_)_10_ | O | 4-(acetylamino)-phenyl |
| **20** |  | (CH_2_)_10_ | O | 4-(1,2,4-triazol-1-yl)-phenyl |
| **21** |  | (CH_2_)_10_ | O | 4-(imidazol-1-yl)-phenyl |
| **22** |  | (CH_2_)_10_ | O | 2-pyridyl |
| **23** |  | (CH_2_)_10_ | O | 3-pyridyl |
| **24** |  | (CH_2_)_10_ | O | 4-pyridyl |
| **25** |  | (CH_2_)_10_ | O | 3-(2-Cl)-pyridyl |
| **26** |  | (CH_2_)_10_ | O | 3-(5-Cl)-pyridyl |
| **27** |  | (CH_2_)_10_ | O | 4-(2-Cl)-pyridyl |
| **28** |  | (CH_2_)_10_ | O | 4-(3-Cl)-pyridyl |
| **29** |  | (CH_2_)_10_ | O | 4-(2-Me)-pyridyl |
| **30** |  | (CH_2_)_10_ | O | 3-(2-Cl-5-F)-pyridyl |
| **31** |  | (CH_2_)_10_ | O | 4-(2,6-di-Me)-pyridyl |
| **32** |  | (CH_2_)_10_ | O | 5-(2-Cl-3-F)-pyridyl |
| **33** |  | (CH_2_)_10_ | O | 4-(6-Me)-pyrimidyl |
| **34** |  | (CH_2_)_10_ | O | 4-(2,6-di-Me)-pyrimidyl |
| **35** |  | (CH_2_)_10_ | O | 4-(2-(trifluoromethyl)-pyrimidyl |
| **36** | Mito-Q type | (CH_2_)_10_ | O | CO(CH_2_)_3_P^+^Ph_3_ Br^-^ |

**Group 2:** CoQ_10_-type quinones

**37 – 42 43 – 44**

| **Cpd** | **Compound name** | **R1** | **R2** | **R3** | **n** | **R4** | **R5** |
| --- | --- | --- | --- | --- | --- | --- | --- |
| **37** | 1,4-benzoquinone | H | H | H | 0 | H | - |
| **38** | 2,3,5-trimethylbenzo-1,4,-quinone | CH_3_ | CH_3_ | CH_3_ | 0 | H | - |
| **39** | CoQ1 | OCH_3_ | OCH_3_ | CH_3_ | 1 | H | - |
| **40** | CoQ2 | OCH_3_ | OCH_3_ | CH_3_ | 2 | H | - |
| **41** | CoQ4 | OCH_3_ | OCH_3_ | CH_3_ | 4 | H | - |
| **42** | CoQ10 | OCH_3_ | OCH_3_ | CH_3_ | 10 | H | - |
| **43** | CoQ0 | OCH_3_ | OCH_3_ | CH_3_ | - | - | CH_3_ |
| **44** | 2-allyl-6-methoxybenzo-1,4,-quinone | OCH_3_ | H | H | - | - | CH_2_CH=CH_2_ |

**Group 3:** Vitamin E-type compounds

| **Cpd** | **Compound name / type** | **Chemical structure** |
| --- | --- | --- |
| **45** | alpha-tocopherol quinone (Edison A-0001) |  |
| **46** | alpha-tocopherol |  |
| **47** | alpha-tocotrienol quinone (Edison EPI-743) |  |
| **48** | alpha-tocotrienol |  |

**Group 4:** Vitamin K-type compounds

| **Cpd** | **Compound name / type** | **Chemical structure** |
| --- | --- | --- |
| **49** | vitamin K1 (phylloquinone) |  |
| **50** | vitamin K2 (menatetrenone) |  |
| **51** | menadione |  |
| **52** | beta-lapachone |  |

**Group 5**

| **Cpd** | **R1** | **R2** | **R3** | **R4** |
| --- | --- | --- | --- | --- |
| **53** | F | H | H | H |
| **54** | CH_3_ | H | H | H |
| **55** | CH_3_ | CH_3_ | H | H |
| **56** | H | H | CH_3_ | CH_3_ |
| **57** | H | H | -CH_2_CH_2_- | |

**Group 6**

| **Cpd** | **X** | **R1** | **R2** |
| --- | --- | --- | --- |
| **58** | (CH_2_)_4_C≡C(CH_2_)_3_C≡CCH_2_ | CH_3_ | OH |
| **59** | (CH_2_)_10_ | H | H |
| **60** | (CH_2_)_10_ | CH_3_ | OH |

**Group 7**

| **Cpd** | **R1** | **X** | **Y** | **R3** |
| --- | --- | --- | --- | --- |
| **61** | OH | NH | (CH_2_)_5_ | OH |
| **62** | OH | NH | (CH_2_)_10_ | OH |
| **63** | OCH_3_ | NH | (CH_2_)_5_ | OH |
| **64** | OCH_3_ | NH | (CH_2_)_10_ | OH |
| **65** | OCH_3_ | O | (CH_2_)_9_ | OH |
| **66** | NH(CH_2_)_5_OH | NH | (CH_2_)_5_ | OH |

**Group 8**

| **Cpd** | **R1** | **R2** | **Y** | **R3** |
| --- | --- | --- | --- | --- |
| **67** | OH | OH | (CH_2_)_11_ | H |
| **68** | OCH_3_ | OCH_3_ | (CH_2_)_11_ | H |

**Group 9:** Oligopeptide-idebenone conjugates

| **Cpd** | **Chemical structure** |
| --- | --- |
| **69** |  |
| **70** |  |
